# Endodontic Treatment Outcomes in Cone Beam Computed Tomography Images—Assessment of the Diagnostic Accuracy of AI

**DOI:** 10.3390/jcm13144116

**Published:** 2024-07-14

**Authors:** Wojciech Kazimierczak, Natalia Kazimierczak, Julien Issa, Róża Wajer, Adrian Wajer, Sandra Kalka, Zbigniew Serafin

**Affiliations:** 1 Kazimierczak Private Medical Practice, Dworcowa 13/u6a, 85-009 Bydgoszcz, Poland; 2 Department of Radiology and Diagnostic Imaging, University Hospital No. 1 in Bydgoszcz, Marii Skłodowskiej Curie 9, 85-094 Bydgoszcz, Poland; 3 Department of Radiology and Diagnostic Imaging, Collegium Medicum, Nicolaus Copernicus University in Torun, Jagiellońska 13–15, 85-067 Bydgoszcz, Poland; 4 Chair of Practical Clinical Dentistry, Department of Diagnostics, Poznań University of Medical Sciences, 61-701 Poznań, Poland; 5 Dental Primus, Poznańska 18, 88-100 Inowrocław, Poland

**Keywords:** artificial intelligence (AI), automatic detection, diagnosis, diagnostic test accuracy, CBCT, cone beam computed tomography, endodontic treatment

## Abstract

**Background/Objectives:** The aim of this study was to assess the diagnostic accuracy of the AI-driven platform Diagnocat for evaluating endodontic treatment outcomes using cone beam computed tomography (CBCT) images. **Methods:** A total of 55 consecutive patients (15 males and 40 females, aged 12–70 years) referred for CBCT imaging were included. CBCT images were analyzed using Diagnocat’s AI platform, which assessed parameters such as the probability of filling, adequate obturation, adequate density, overfilling, voids in filling, short filling, and root canal number. The images were also evaluated by two experienced human readers. Diagnostic accuracy metrics (accuracy, precision, recall, and F1 score) were assessed and compared to the readers’ consensus, which served as the reference standard. **Results:** The AI platform demonstrated high diagnostic accuracy for most parameters, with perfect scores for the probability of filling (accuracy, precision, recall, F1 = 100%). Adequate obturation showed moderate performance (accuracy = 84.1%, precision = 66.7%, recall = 92.3%, and F1 = 77.4%). Adequate density (accuracy = 95.5%, precision, recall, and F1 = 97.2%), overfilling (accuracy = 95.5%, precision = 86.7%, recall = 100%, and F1 = 92.9%), and short fillings (accuracy = 95.5%, precision = 100%, recall = 86.7%, and F1 = 92.9%) also exhibited strong performance. The performance of AI for voids in filling detection (accuracy = 88.6%, precision = 88.9%, recall = 66.7%, and F1 = 76.2%) highlighted areas for improvement. **Conclusions:** The AI platform Diagnocat showed high diagnostic accuracy in evaluating endodontic treatment outcomes using CBCT images, indicating its potential as a valuable tool in dental radiology.

## 1. Introduction

The main goal of endodontic treatment is to eradicate diseased pulp tissue and foster an environment that supports the healing of periapical tissues, ultimately preventing the development of apical periodontitis. Damaged teeth can be preserved by extracting diseased tissue, sealing the canal system, and repairing the coronal tooth structure. This preservation is essential for maintaining the integrity of the dental arch, as well as for aesthetic and functional purposes, which are often the main concerns of patients. Despite the vast body of scientific literature discussing the features of successful endodontic treatment, there is significant variability in study protocols and data [1].

Radiographic success in endodontic treatment is primarily determined by the absence of periapical radiolucency and the presence of a well-sealed root canal filling. The success of endodontic treatment is largely influenced by the quality of the root canal filling. A well-sealed root canal filling is essential for preventing microbial infiltration and subsequent infection. The technical quality of the root canal filling, including its length and density, is often assessed radiographically to determine the success of the treatment. Studies have shown that deficiencies in the extent and density of root canal filling are associated with a higher failure rate [2,3]. Moreover, radiographic imaging is an important tool in postendodontic treatment evaluation. The absence of pathologic radiolucency in follow-up radiographs is a key indicator of successful endodontic therapy [4].

Radiographic assessment of endodontic treatment has a long history, dating back to Dr. Edmund Kells. In 1899, he was the first to determine the working length in endodontics using dental X-rays [2,3]. Periapical radiography (PR) has remained the primary imaging modality and an indispensable tool in endodontic practices for many decades. However, it has inherent limitations, such as the difficulty of assessing buccolingual dimensions and distinguishing between inflammatory tissues and healed fibrous scar tissue [4]. Orthopantomograms (OPGs), which cover the maxilla, mandible, all teeth, and temporomandibular joints, may be indicated in endodontic assessments in cases of large periapical lesions that might extend beyond the imaging capabilities of PRs. However, compared with PRs, OPGs have been shown to have significantly lower diagnostic accuracy in detecting periapical lesions [5]. Surrounding structures and complex anatomy can render interpretation of planar images difficult. Both PR and OPGs have significant inherent limitations due to the two-dimensional (2D) representation of three-dimensional (3D) structures. These limitations include geometric distortions, inconsistent magnification, overlapping structures, and anatomical noise [6,7,8]. The response to these problems has been the introduction of cone beam computed tomography (CBCT), which overcomes these limitations, enabling accurate 3D visualization of multiplanar details of oral structures. A spatial resolution of less than 0.1 mm allows for precise diagnosis and measurements in endodontic evaluation [9,10].

The recent rise in the utilization of artificial intelligence (AI) tools in medicine has extended to dentistry, particularly in the field of dentomaxillofacial radiology [11]. AI has increasingly been integrated into endodontic treatment assessment, offering potential improvements in diagnostic accuracy, treatment planning, and prognostic predictions [12,13]. AI models, particularly convolutional neural networks (CNNs) and artificial neural networks (ANNs), have shown high accuracy in detecting periapical lesions (PLs), vertical root fractures, and root canal system anatomy [12,13]. Moreover, AI programs have been shown to expedite the clinical decision-making process, reducing the time required for X-ray image analysis and clinical data interpretation [14]. Furthermore, AI models have demonstrated the ability to predict postoperative pain, treatment efficacy, and case difficulty, aiding in comprehensive treatment planning [15,16]. Diagnostic, Ltd. (San Francisco, CA, USA) has created a web-based, commercially available system employing CNNs to deliver precise and comprehensive dental diagnostics. This system has been trained on more than 35,000 dental radiographs to boost its diagnostic capabilities. This system allows for detailed evaluation of endodontically treated teeth, theoretically facilitating prompt and accurate diagnoses.

This preliminary study aimed to evaluate the diagnostic accuracy of Diagnocat, an AI-driven platform, in assessing radiographic endodontic treatment outcomes using CBCT images.

## 2. Materials and Methods

The study adhered to the principles of the Declaration of Helsinki and received approval from the Ethics Committee of Collegium Medicum, Nicolaus Copernicus University in Torun, Poland (protocol No. KB 227/2023, 10.04.2023), for research involving human participants.

### 2.1. Patients

The study involved a total of 55 consecutive patients (15 males and 40 females, aged between 12 and 70 years) who were referred for CBCT imaging at a private dental center. All patients were referred for imaging by orthodontists and dental surgeons between January and September 2023. The primary clinical indications for the CBCT scans included suspicion of PLs in the orthopantomograms (OPGs) and the presence of an impacted tooth. X-ray images with severe motion artifacts altering the quality of the image were excluded, as were scans with a field of view (FOV) that did not cover the periapical region of all presented teeth.

### 2.2. Image Acquisition and Postprocessing

The reading sessions were conducted on a dedicated console utilizing iRYS Viewer software version 6.2 (MyRay, Imola, Italy). All images were examined on a RadiForce MX243W (Eizo, Hakusan, Japan) monitor, which is certified for medical use. The following imaging settings were used during CBCT analysis: window width, 1048 HU; center, 4096 HU. All the CBCT images were reconstructed with a slice thickness of 0.3 mm. The readers were free to adjust the image window settings.

### 2.3. AI Evaluation

CBCT images of each of the included patients were manually uploaded to the cloud-based commercially available platform Diagnocat. The AI software automatically provided separate reports for both imaging modalities with estimated probabilities (0–100%) of changes occurring.

CBCT images of each patient were manually uploaded to the cloud-based commercial platform Diagnocat. The AI software then automatically generated reports indicating the estimated probability (ranging from 0 to 100%) of the observed changes.

In the case of endodontic treatment, the program assessed the following endodontic parameters:

1. Probability of filling
2. Adequate obturation
3. Adequate density
4. Overfilling
5. Voids in filling
6. Short filling
7. Root canal number

A probability of 50% of the condition occurring was regarded as a positive diagnosis.

### 2.4. Human Reader Evaluation

The images were assessed by two readers, a dentist and a radiology specialist, each with more than five years of experience in dentomaxillofacial CBCT evaluation. Both readers were unaware of each other’s assessments or of the AI software’s evaluation results. The presence of endodontic treatment was recorded for each tooth. The features listed in the AI reports were assessed, and the following parameters were considered:

1. Probability of filling—evaluation of the presence of radiopaque material within the tooth canals.
2. Adequate obturation—assessment of the extent of the filling material up to the apex of the root, verification of material density and consistency through the canal.
3. Adequate density—evaluation of the radiopacity and homogeneity of the filling material and identification of areas with lower density indicating voids or inadequate filling.
4. Overfilling—examination of any filling material extending beyond the apex of the tooth.
5. Voids in filling—identification of radiolucent areas within the filling that indicate the presence of voids.
6. Short filling—assessment of the extent of the filling material if it falls short of the tooth apex.

The readers were blinded to the results of the AI evaluations. After the separate reading sessions, they jointly evaluated all the images and reached a consensus, which was considered the reference standard.

### 2.5. Statistical Evaluation

The accuracy, precision, recall, and F1 score were calculated to assess the accuracy of the AI results. The diagnostic accuracy calculations were made according to the method presented by Hicks et al. [17]. The concordance of the quantitative measures between the two methods was assessed with an intraclass correlation coefficient of type 3 (according to Shrout and Fleiss). The means, standard deviations, medians, quartiles, and ranges of the quantitative variables are shown. For qualitative variables, absolute and relative frequencies (N and %) were reported. The *p* value used for statistical significance in this study was set at <0.05. All the analyses were conducted in R software, version 4.4.0.

## 3. Results

### 3.1. Patients

The study included 55 sets of CBCT images from 55 patients, with participants having a mean age of 44.53 years, ranging from 12 to 70 years. The sample comprised 40 females with an average age of 42.83 years and 15 males with an average age of 50.73 years. In total, 1330 teeth were used in the calculations.

### 3.2. Diagnostic Accuracy Parameters

Table 1 presents the diagnostic performance metrics of AI compared to reader evaluations across CBCT images.

**Figure 1:**
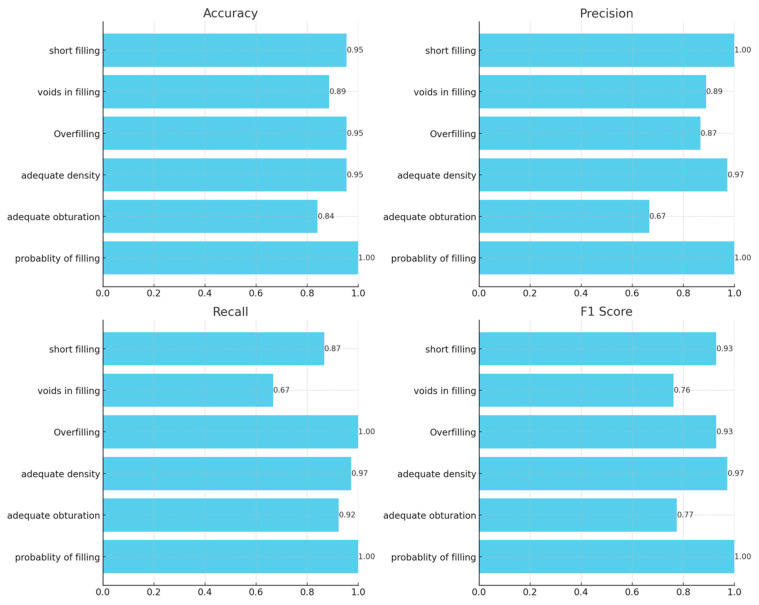
Diagnostic accuracy metrics of the selected parameters of endodontic treatment.

**Figure 2:**
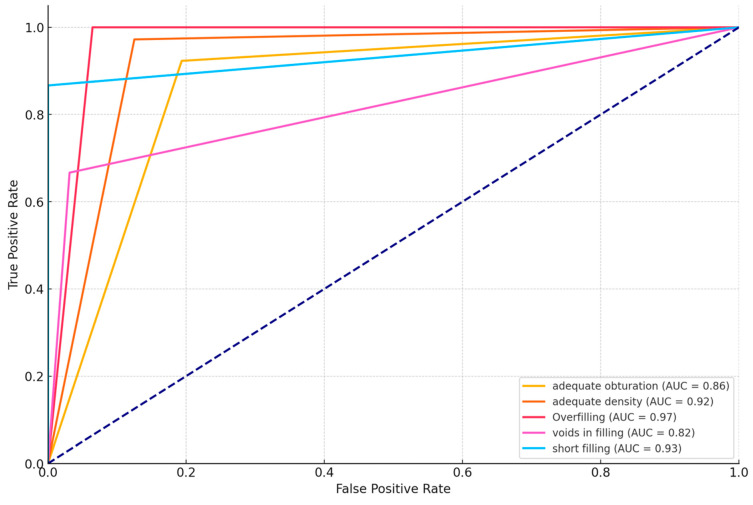
ROC curves for all the assessed endodontic treatment parameters.

Diagnocat demonstrated exceptional performance in predicting the probability of filling, achieving perfect scores across all the metrics: 100% accuracy, precision, recall, and F1 score. Adequate obturation metrics show more variation, with the AI algorithm achieving an accuracy of 84.1% and an F1 score of 77.4%. Adequate density results were highly consistent with the readers’ consensus, with an accuracy of 95.5% and an F1 score of 97.2%. Overfilling and short-filling assessments showed high, exceeding 90% accuracy. However, voids in fillings detection results showed the platform’s slightly lower accuracy of 88.6% and an F1 score of 76.2%. An additional analysis was conducted to determine whether there were specific teeth or sections where the evaluation was more limited. The results indicated that posterior teeth showed slightly lower accuracy, possibly due to their complex root anatomy. However, due to limited evidence (only a few misdiagnoses), we cannot draw any conclusions. Figure 1 graphically presents the results of the diagnostic accuracy assessment. Receiver operating characteristic (ROC) curves for all the assessed parameters are presented in Figure 2. The calculated *p* values for the ROC curves were all <0.05, indicating statistical significance.

The results of the concordance of the number of root canals reported by the human reader and AI software in the CBCT assessment exhibited excellent agreement (Table 2).

## 4. Discussion

The results of our study highlight the considerable potential of AI in improving the diagnostic accuracy of endodontic treatment outcome evaluations using CBCT images. Our findings demonstrate the effectiveness of the Diagnocat AI-powered platform in precisely evaluating several critical radiographic features of endodontic treatment. Our results showed that the AI system demonstrated excellent diagnostic accuracy for most of the assessed root canal features, with slightly lower accuracy in void filling assessment.

Accurate assessment of endodontic treatment outcomes is crucial for ensuring long-term dental and oral health. CBCT offers high-resolution, 3D digital radiographic scans that can be further digitally adjusted to enhance the visibility of anatomical structures [18]. This makes CBCT a useful tool for evaluating the quality of endodontic treatments. Currently, CBCT is used in endodontics for numerous purposes, including pre- and postprocedural assessments. It allows for detailed diagnosis of existing dental conditions and preoperative treatment, evaluation of root resorption, and treatment planning, including preparation of endodontic guides and evaluation of outcomes [19]. CBCT imaging has exhibited greater sensitivity and specificity in identifying PLs than traditional periapical or panoramic radiographs [4]. CBCT offers superior validity and reliability compared to intraoral radiography for diagnosing and managing endodontic complications [20,21]. It provides undistorted three-dimensional images, allowing for better assessment of periapical disease, root fractures, root canal anatomy, and alveolar bone topography [22,23]. An important study by Lo Giudice et al. [24] evaluated the diagnostic accuracy of PR and CBCT images in assessing endodontic treatment outcomes. The authors analyzed 101 patients with previous endodontic treatments who underwent both PR and CBCT imaging. CBCT proved to be a more accurate diagnostic tool for endodontic problems than conventional intraoral radiographs, detecting important radiological signs not always visible in periapical X-rays, particularly short fillings and untreated canals (up to 30.6%). A recent study by Keerthana et al. [25] evaluated PR and CBCT images in the assessment of the endodontic status of 112 teeth. This study showed that CBCT has greater diagnostic accuracy in diagnosing complex endodontic pathoses than PR. However, CBCT failed to detect apicomarginal bone defects in 33% of teeth, and limited-FOV CBCT should be considered for selective cases where the PR has diagnostic ambiguity.

Our study showed that Diagnocat exhibited exceptional performance in detecting the probability of root canal filling, with perfect scores across accuracy, precision, recall, and F1 metrics. This indicates that AI has a strong ability to identify the presence of radiopaque material within tooth canals, a critical aspect of endodontic treatment evaluation. For adequate obturation, the algorithm showed moderate performance, with an accuracy of 84.1% and an F1 score of 77.4%. The lower diagnostic parameters of Diagnocat in this area were due to incorrect diagnoses such as short filling, overfilling, and voids in filling. False-negative assessments in these categories resulted in false-positive adequate obturation diagnoses. The observed limitations in the accuracy of ‘adequate obturation’ and the recall of ‘filling voids’ may be attributed to the inherent challenges in detecting voids within the filling material and the variability in the radiopacity of different filling materials used. Additionally, the complex anatomy of certain teeth can obscure clear visualization of these parameters, leading to discrepancies in the diagnostic performance of AI.

**Figure 3:**
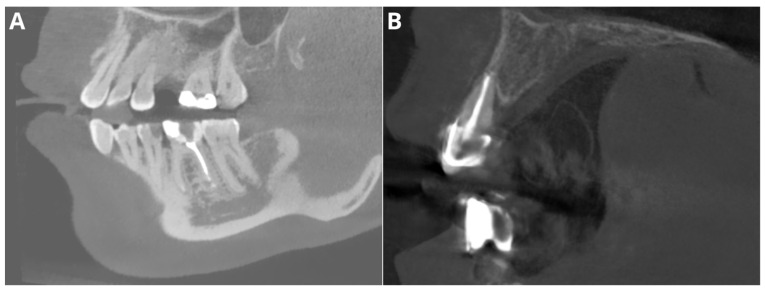
Two of the false negative diagnoses involved overfilling. (**A**)—tooth 46; (**B**)—tooth 12.

**Figure 4:**
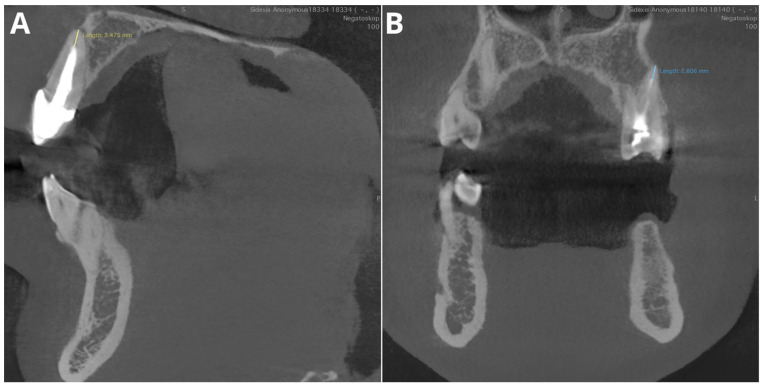
Two of the false negative short-filling diagnoses. (**A**)—tooth 12, filling 3.5 mm from the apex; (**B**)—tooth 47, filling 2.8 mm from the apex.

The performance for adequate density was notably high, with an accuracy of 95.5%, a precision of 97.2%, a recall of 97.2%, and an F1 score of 97.2%. Considering that this parameter relies on the subjective evaluation of the readers, we assume that the tested AI program exhibited perfect agreement with the readers’ assessments. Diagnosis revealed overfilling with high accuracy (95.5%), precision (86.7%), recall (100%), and F1 score (92.9%) on the CBCT images. An evaluation of the misdiagnoses revealed that all cases were very subtle (Figure 3). In contrast, Orhan et al. [26] reported a sensitivity of 66.7% for Diagnocat’s overfilling detection on panoramic images. These differences may stem both from the program’s algorithms’ improvements over time and from the different imaging modalities. Moreover, Diagnocat showed exceptional diagnostic accuracy in short-term assessments, with close to 100% accuracy. Figure 4 presents two of the false-negative diagnoses. The detection of voids by the AI program achieved lower performance than other parameters in our study, with 88.6% accuracy, 88.9% precision, 66.7% recall, and 76.2% F1 score. However, Orhan et al. reported a much lower sensitivity of 23.3% for void detection on panoramic images [26]. This discrepancy could be attributed to the 3D imaging provided by CBCT compared to the 2D panoramic images used in Orhan’s study. Our study revealed high accuracy and precision for diagnosing short fillings (95.5% and 100%, respectively), along with a recall of 86.7% and an F1 score of 92.9%. However, Orhan et al. reported a lower sensitivity of 70% [26].

Zadrożny et al. reported unsatisfactory diagnostic accuracy metrics similar to those of Orhan in the assessment of panoramic images for endodontic treatment outcomes [27]. Although the authors reported the program’s high sensitivity for endodontic treatment detection (87.2%), the system showed much lower sensitivity for over- and underfilled canals, ranging between 45.5% and 60.9%. However, the study evaluated only 30 panoramic radiographs. Our recent study evaluated the diagnostic performance of Diagnocat in assessing endodontic treatment results via panoramic radiographs [28]. The AI system exhibited excellent accuracy (90.72%) and an impressive F1 score (95.12%) when identifying the probability of endodontic filling. However, its performance in other categories was inconsistent. The system delivered subpar accuracy and unacceptable F1 scores for evaluating short fillings and voids in fillings, registering only 8.33% and 14.29%, respectively. The accuracy for detecting adequate obturation and density was moderate, at 55.81% and 62.79%, respectively. However, this preliminary study evaluated 55 patients. These results, in our view, highlight the fundamental limitations of using panoramic radiography for endodontic diagnostics, as well as the constraints of the Diagnocat program itself. We have concluded that given that panoramic radiographs are not the preferred imaging modality for endodontic evaluations, we find Diagnocat to be a valuable tool for postendodontic treatment assessments. In our opinion, this discrepancy is primarily due to the inherent limitations of 2D imaging, such as geometric distortions, inconsistent magnification, overlapping structures, and anatomical noise. These factors can obscure diagnostic features and lead to less accurate evaluations. In our opinion, this discrepancy is primarily due to the inherent limitations of 2D imaging, such as geometric distortions, inconsistent magnification, overlapping structures, and anatomical noise. These factors can obscure diagnostic features and lead to less accurate evaluations. Our findings are confirmed by the previously cited works of Orhan and Zadrożny [26,27].

**Figure 5:**
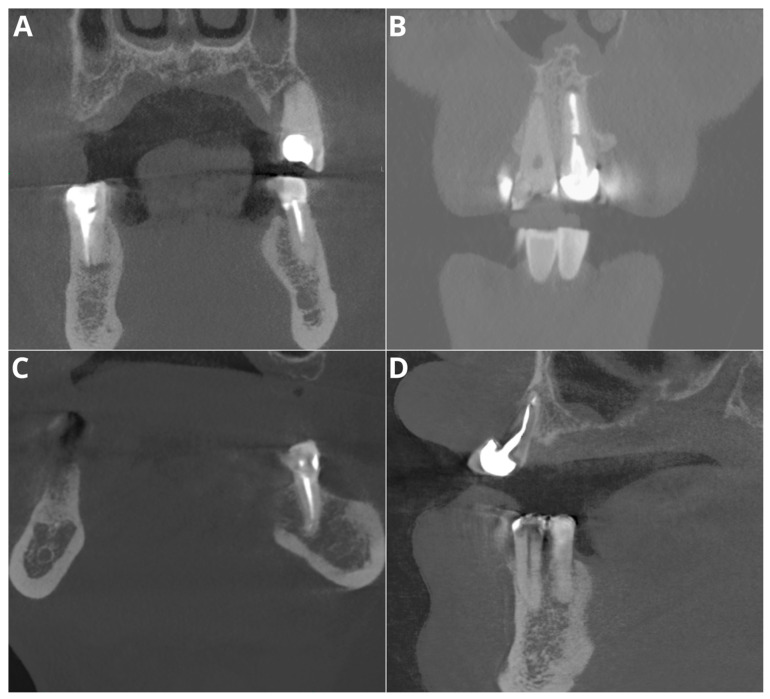
Misdiagnoses in voids in filling feature assessment. (**A**)—tooth 35—false positive; (**B**)—tooth 21—false negative; (**C**)—tooth 34—false negative; (**D**)—tooth 13—false negative.

In general, our results showed excellent diagnostic accuracy for Diagnocat in endodontic treatment evaluation. A detailed investigation during the preparation of figures revealed only one false positive diagnosis among all the evaluated features and teeth. A false positive diagnosis was made in the voids in the filling assessment (shown in Figure 5A). This finding aligns with the findings of other studies showing high specificity in diagnosing this condition, although with slightly lower sensitivity metrics [26,27,29,30,31]. These metrics demonstrate the “conservativeness” of the tested AI program, where at the cost of false negatives, there is no excessive number of false positives. The integration of such AI programs in endodontic diagnostics, particularly with CBCT imaging, presents several clinical advantages. It not only improves diagnostic accuracy but also standardizes the assessment process, thereby minimizing inter- and intraobserver variability. This is particularly beneficial in high-volume practices where consistent and rapid diagnostic evaluations are crucial.

In 2023, a meta-analysis evaluated the influence of root canal filling quality on PL status using CBCT [32]. The authors found a significant association between the results of CBCT quality assessments and PL healing. The study showed that root canal treatment success is directly linked to the apical extent of the filling, which is ideally positioned between 0 and 2 mm short of the apical foramen, and the homogeneity of the filling without any gaps [32]. The use of AI can aid in the detection of treatment deficiencies, such as inadequate obturation, short fillings, or the presence of voids, allowing timely intervention and potentially improving long-term treatment outcomes. The high sensitivity and specificity of AI in detecting these parameters, as demonstrated in this study, underscore its potential utility in routine endodontic practice.

Several studies have evaluated the diagnostic performance of Diagnocat for detecting PLs using various imaging modalities. Orhan et al. [33] performed a study evaluating the performance platform in identifying PLs on CBCT scans. The results showed that Diagnocat presented an impressive sensitivity of 92.8% in this task. Zadrozny et al. assessed the diagnostic performance of Diagnocat in detecting PLs using 30 panoramic radiographs encompassing a total of 805 teeth [27]. The study revealed that the diagnostic sensitivity was relatively low at 30.9%, but the specificity was very high at 98.1%. In another study, Issa et al. examined the accuracy of the diagnostic tool for diagnosing periapical periodontitis in PRs [34]. The results showed that the program had a high sensitivity of 92.3% for identifying PLs and a specificity of 97.87% for recognizing healthy teeth. Additionally, the recorded accuracy and F1 score were 96.66% and 92%, respectively. Our recent study assessed the diagnostic accuracy of Diagnocat for PL detection on OPG and CBC images acquired from one patient cohort [30]. This study involved 49 patients with a total of 1223 teeth whose images were analyzed by both AI and experienced clinicians. The results showed that the AI had a sensitivity of 33.33% and an F1 score of 32.73% for the OPG images, whereas for the CBCT images, the sensitivity was 77.78% with an F1 score of 84.00%. The AI demonstrated over 98% specificity for both the OPG and CBCT images. The results show that the AI was highly sensitive and specific for detecting PLs in CBCT images, but showed lower sensitivity in OPG images.

The sensitivity of different radiographic techniques for detecting endodontic treatment success varies. Studies indicate that CBCT is more accurate than plain film radiography (PFR) and digital PR for identifying small periapical lesions and deficiencies in root canal fillings. A study by Fernandes et al. over a 5-year period revealed that CBCT was more sensitive than conventional imaging modalities for detecting issues with root canal fillings [3]. Moreover, poor treatment outcomes were significantly associated with factors such as root canal curvature, missed canals, and the quality of coronal restoration. While CBCT offers a more precise assessment, traditional methods such as PFR and DPR are still commonly used due to their accessibility and lower cost. Moreover, CBCT is limited by its high ionizing radiation exposure. The joint statement from the American Association of Endodontists and the American Academy of Oral and Maxillofacial Radiology specifies that CBCT imaging should be utilized only in instances where lower-dose conventional radiography or other imaging methods fail to provide an adequate diagnosis [35]. PRs remain the modality of choice for radiographic endodontic evaluation. However, limited-FOV CBCT is preferred for the preoperative assessment of teeth with complex anatomy, patients with nonspecific clinical signs associated with previously treated endodontic teeth, nonhealing endodontically treated teeth, and endodontic treatment complications. The authors concluded that the use of limited-FOV CBCT should be considered on a case-by-case basis, always weighing the risks associated with ionizing radiation exposure. Moreover, small-FOV CBCT images result in a lower radiation dose, smaller voxel size, higher resolution, and reduced noise and scatter [20]. Therefore, the routine use of large-FOV CBCT as a basic diagnostic modality is unjustified, especially in children who are more sensitive to radiation. Consequently, every CBCT scan should be justified and tailored to meet the specific clinical scenario, following the basic principles of radiation protection [36,37]. The indications for CBCT scans covering all patients’ teeth in our study sample were heterogeneous, including the presence of impacted teeth, PLs on both sides of the dental arches, detection of bony changes, and preoperative assessment for implant procedures. Therefore, all the CBCT scans in our sample were justified.

A systematic review by Korabari et al. [12] assessed the diagnostic and prognostic potential of AI in endodontic treatment and indicated that AI could significantly enhance treatment planning and improve the success rates of endodontic procedures. This study highlighted the advantages of AI in various clinical tasks, including detecting root fractures and PLs, determining working lengths, tracing the apical foramen, and assessing root morphology. Similar results were shown in a paper by Ramezanzade et al. [13], where the authors demonstrated the high effectiveness of AI in specific endodontic tasks: PL and vertical root fracture detection, root canal morphology prediction, apical foramen localization, endodontic retreatment prediction, and tooth segmentation. However, the reviews did not delve into the effectiveness of AI in the radiographic evaluation of endodontic treatment outcomes. Despite the encouraging findings, the authors emphasized the need for extensive research before fully integrating AI systems into routine practice and noted a high risk of bias in the evaluated studies. These findings are consistent with broader trends in radiology and other medical fields, suggesting the need for caution against the indiscriminate and insufficiently studied deployment of AI systems [38,39].

Despite the promising results, this study has limitations. The study sample size was relatively small, although the total number of teeth included allowed for statistically significant conclusions to be drawn. Another limitation was that the evaluation was confined to a specific AI platform. Future research should encompass larger, more diverse populations and compare multiple AI systems to further validate these findings. Furthermore, the reference standard set by the readers’ consensus might be biased due to errors in the assessments. However, this is an inherent characteristic of studies evaluating diagnostic accuracy. Additionally, the retrospective nature of the study may have affected the outcomes. Prospective studies with standardized protocols are recommended to address these limitations. Additionally, while AI showed high accuracy in most parameters, its lower recall rates for detecting voids and short fillings highlight areas needing improvement. Future advancements in AI algorithms should focus on enhancing the sensitivity and specificity of these critical diagnostic features.

## 5. Conclusions

In conclusion, this study validated the high diagnostic accuracy of the Diagnocat AI platform for evaluating endodontic treatment outcomes using CBCT images. The tested AI platform demonstrated high accuracy, exceeding 95.5%, in the assessment of most endodontic treatment features. However, it showed slightly lower accuracy, at 88.6%, in the assessment of voids in fillings. The results indicate that AI can be used to analyze complex 3D imaging data effectively, offering a reliable tool for endodontic assessment. However, further research with larger sample sizes is necessary before implementing the tested AI platform in routine daily practice.

## Figures and Tables

**Table 1:** Diagnostic accuracy metrics of the tested AI platform for endodontic treatment outcome assessment.

Parameter	Accuracy	Precision	Recall	F1 Score
Probability of filling	100%	100%	100%	100%
Adequate obturation	84.1%	66.7%	92,3%	77.4%
Adequate density	95.5%	97.2%	97.2%	97.2%
Overfilling	95.5%	86.7%	100%	92.9%
Voids in filling	88.6%	88.9%	66.7%	76.2%
Short fillings	95.5%	100%	86.7%	92.9%

**Table 2:** Concordance analysis between the AI program and human readers’ consensus on the number of root canals in evaluated endodontically treated teeth.

Parameter	ICC	95% CI		Agreement (Cicchetti)	Agreement (Koo and Li)
Root canals number	0.958	0.941	0.971	Excellent	Excellent

CI—Confidence intervals.

## Data Availability

Data are available upon request.
